# Total antioxidant intake and prostate cancer in the Cancer of the Prostate in Sweden (CAPS) study. A case control study

**DOI:** 10.1186/s12885-016-2486-8

**Published:** 2016-07-11

**Authors:** Kjell M. Russnes, Elisabeth Möller, Kathryn M. Wilson, Monica Carlsen, Rune Blomhoff, Sigbjørn Smeland, Hans-Olov Adami, Henrik Grönberg, Lorelei A. Mucci, Katarina Bälter

**Affiliations:** Oslo University Hospital, Clinic of Cancer, Surgery and Transplantation, Montebello, 0380 Oslo Norway; Department of Medical Epidemiology and Biostatistics, Karolinska Institute, 17177 Stockholm, Sweden; Department of Nutrition, Institute for Basic Medical Sciences, University of Oslo, Sognsvannsveien 9, 0372 Oslo, Norway; Department of Epidemiology, Harvard School of Public Health, 677 Huntington Avenue, Boston, MA 02115 USA; Channing Laboratory, 181 Longwood Ave, Boston, MA 02115 USA

**Keywords:** Antioxidants, Prostate cancer, Coffee, Dietary supplement

## Abstract

**Background:**

The total intake of dietary antioxidants may reduce prostate cancer risk but available data are sparse and the possible role of supplements unclear. We investigated the potential association between total and dietary antioxidant intake and prostate cancer in a Swedish population.

**Methods:**

We used FFQ data from 1499 cases and 1112 controls in the population based case–control study Cancer of the Prostate in Sweden (CAPS). The ferric reducing antioxidant potential (FRAP) assay was used to assess the total antioxidant capacity (TAC) of diet and supplements. We calculated odds ratios (ORs) for the risk of prostate cancer across quintiles of antioxidant intake from all foods, from fruit and vegetables only, and from dietary supplements using unconditional logistic regression.

**Results:**

Coffee comprised 62 % of the dietary antioxidant intake, tea 4 %, berries 4 %, chocolate 2 %, and boiled potatoes 2 %. In total 19 % and 13 % of the population took multivitamins and supplemental Vitamin C respectively, on a regular basis. Antioxidant intake from all foods and from fruits and vegetables separately measured by the FRAP assay was not associated with prostate cancer risk. For antioxidant intake from supplements we found a positive association with total, advanced, localized, high grade and low grade prostate cancer in those above median supplemental TAC intake of users compared to non-users (Adjusted ORs for total prostate cancer: 1.37, 95 % CI 1.08–1.73, advanced: 1.51, 95 % CI 1.11–2.06, localized: 1.36. 95 % CI 1.06–1.76, high grade 1.60, 95 % CI 1.06–2.40, low grade 1.36, 95 % CI 1.03–1.81). A high intake of coffee (≥6 cups/day) was associated with a possible risk reduction of fatal and significantly with reduced risk for high grade prostate cancer, adjusted OR: 0.45 (95 % CI: 0.22–0.90), whereas a high intake of chocolate was positively associated with risk of total, advanced, localized and low grade disease (adjusted OR for total: 1.43, 95 % CI 1.12–1.82, advanced: 1.40, 95 % CI 1.01–1.96, localized: 1.43, 95 % CI 1.08–1.88, low-grade: 1.41, 95 % CI 1.03–1.93).

**Conclusions:**

Total antioxidant intake from diet was not associated with prostate cancer risk. Supplement use may be associated with greater risk of disease.

**Electronic supplementary material:**

The online version of this article (doi:10.1186/s12885-016-2486-8) contains supplementary material, which is available to authorized users.

## Background

The potential cancer protective effect of diets rich in antioxidants has been extensively studied in relation to different cancer sites. Most observational studies and intervention, have examined the effect of either single or a few antioxidant compounds, or a combined score of redox active compounds, but no consistent protective effect of antioxidant intake on prostate cancer has been shown [1–5].

Because naturally occurring antioxidants in, for example, fruits and vegetables work in a network of redox active compounds, it could be more informative and potentially reduce confounding by other effects of redox active compounds to study the total intake of antioxidants. Several methods have been developed to quantify total dietary antioxidant content [6]. We used the Ferric-reducing ability of plasma (FRAP) assay, a fast, easy to use, and reproducible method to assess total antioxidant content (TAC) in foods, beverages and supplements [7]. We recently reported that total antioxidant intake from diet reduced risk of total, lethal and advanced prostate cancer in the Health Professionals Follow-up Study [8]. We found a weak protective effect of total antioxidant intake from diet, as well as for coffee, one of the largest contributors to antioxidant intake. On the other hand, total antioxidant intake from supplements increased risk for lethal and advanced prostate cancer [6, 8].

In the current study, we examined the association between total antioxidant capacity (TAC) from diet and supplements and prostate cancer in a large population based case–control study of prostate cancer in Sweden. This study points out some differences in antioxidant intake between a Scandinavian and a US population, whilst the large number of cases allowed analyses of subgroups of prostate cancer.

## Methods

### Study population

The Cancer of the Prostate in Sweden (CAPS) study is a population-based case–control study of prostate cancer as described previously [9, 10]. Cases from four of the six regional cancer registries in Sweden were recruited through treating physicians in 2001 and 2002. After approval from patients to participate, they were mailed a letter including consent form and included in the study when they filled out a self-administered questionnaire about lifestyle factors (including diet) and family history or donated blood samples which were returned to the study administration. The cancer was histopathologically or cytologically verified. Disease-related information, such as TNM (tumor, node, metastasis) status, clinical stage, Gleason score, and serum prostate-specific antigen (PSA) level at diagnosis was obtained from the National Prostate Cancer Registry and was available for 95 % of the cases. Controls were randomly selected from the Swedish population registry, identified by personal identification number, and frequency-matched to cases by age in 5-year age categories and by region of residence of the cases. When controls had been identified, they were contacted by mail, receiving a letter describing the study. A few weeks later they received the same letter with self-administered questionnaire and equipment for blood sampling as the cases. Linking control subjects with the National Cancer Registry identified potential control subjects with previous prostate cancer history. who were excluded. Of 1895 invited prostate cancer cases, 1499 (79 %) completed the detailed baseline questionnaire about lifestyle and health. Average time between date of diagnosis and date when the questionnaire was sent was 5 months. Of the 1684 invited controls, 1130 (67 %) completed the questionnaire. All participants gave informed consent at the time of enrollment in the study. The ethics committees at Karolinska Institutet and Umeå University in Sweden approved the study.

### Dietary assessment

Dietary data were collected as part of the questionnaire. All participants completed a validated, self-administered 109-item food frequency questionnaire (FFQ) that assessed the participants’ frequency of consumption of foods and beverages over the previous 12 months. The questionnaire included ten additional questions on dietary supplements. This included information about frequency, dosage, type of supplement and duration of use. A shorter version of the FFQ had been validated earlier against weighed food records among women, and found correlations among the major contributors to TAC ranging from 0.32 to 0.71 [11]. Each FFQ item was assigned a FRAP value based on the average value of a limited number of variants of each food item, specified in the Antioxidant Food Table [7, 12] and foods specifically analyzed for this study (Additional file 1). For combined items such as berries, we used pre-estimated weighting of the individual sub-items (raspberries, blueberries etc.) to calculate a combined FRAP value. When an FFQ item did not have a specified FRAP value, the value was imputed based on knowledge of foods and beverages with similar antioxidant profiles. FRAP values were assigned to supplements in the same manner. All analyses of FRAP were performed at the Institute of Nutrition Research, University of Oslo.

To calculate each participant’s total antioxidant capacity intake (TAC), the frequency of consumption of each item was multiplied by its FRAP value and summed across all items consumed. Three exposure variables for TAC intake were created: 1) Dietary TAC from all foods and beverages; 2) TAC from fruit and vegetables only; and 3) TAC from dietary supplements. We used data from the Swedish National Food Administration to calculate total energy intake and intake of nutrients based on the questionnaire data. TAC and nutrient intakes were energy-adjusted using the residual method [8, 13]. We also examined the intake of the main contributors to TAC and their relation to prostate cancer risk.

### Statistical analysis

Participants were divided into quintiles of TAC intake from all foods and from fruit and vegetables giving five levels of intake for these exposure variables. In addition we wanted to test whether the extreme low and high intakes of TAC were associated with disease and performed the calculation based on decile distribution of TAC intake. For TAC from supplements, the non-users comprised the referent group, and the users were categorized at the median intake, giving three levels of intake. This was due to the relatively large number of non-supplement users in this population. We used unconditional logistic regression models with indicator variables for each level of TAC intake, and for each intake category of the main contributors to TAC. Age group and region, matching factors in the study, were included in all models. The fully adjusted models also included: smoking status (never, former, current), BMI (<20, 20–22.5, 22.5–<25, 25–<27.5, 27.5–<30, >30 kg/m^2^), education (0–9 years, 10–12 years, 13+ yrs), and total energy intake (quartiles). Other potential confounders were tested in the model, including: civil/marital status, employment status, family history of prostate cancer, physical activity, as well as intake of alpha-linolenic acid, vitamin-D, calcium, phytoestrogens, red meat, fish and dairy products. None of these were included in the final models as they had no or little effect on the effect estimates or precision.

To test for dose–response trends across the levels of TAC exposures and categories of TAC contributors, we modeled all exposure variables as continuous variables using the median intake in each quintile, decile or category of intake. Analyses of the major contributors to dietary TAC (coffee, tea, berries, chocolate and boiled potatoes) were mutually adjusted, in addition to including the factors in the original TAC model, as well as adjusting for zinc and calcium intake. Similarly, the models for major contributors to supplemental TAC (Vitamin C supplements and multivitamins) were mutually adjusted, and included the same factors as the models for major contributors to dietary TAC. The analysis of intake of TAC and prostate cancer were also performed in subgroups as never smokers and ever smokers.

Advanced prostate cancer was defined as cancer with capsule penetration or seminal vesicle infiltration (T3), invasion of adjacent organs (T4), metastasis to lymph nodes (N+) or distant organs (M1) at the time of diagnosis, or prostate cancer death during follow-up through June 2009. Fatal cases, a subgroup of advanced cases, were defined as participants that died from the disease during follow-up. Localized cases were those with T1 and T2 tumors and no metastases (N0/M0) at the time of diagnosis. Since advanced and lethal disease cases would include cases that died during follow-up, the categories localized and advanced are not mutually exclusive, and therefore the sum of advanced and localized exceeding the total number of cases. High grade cases included those with Gleason score 8–10, and low grade cases included cases with Gleason score 2–6. Gleason score 7 cases were not included in either high-grade or low-grade disease because of the heterogeneity of these tumors, and the different outcomes seen for Gleason 3 + 4 compared to Gleason 4 + 3 [6, 8, 14].

## Results

### Descriptive data

The main contributors to dietary TAC intake in the study population were coffee (62 %), tea (4 %), berries (4 %), chocolate (2 %) and boiled potatoes (2 %). Mean intake of TAC from diet was similar among controls and cases, 22.2 and 22.6 mmol/day respectively, and TAC from fruit and vegetables was 3.5 mmol/day among both cases and controls (Table 1). Among controls the mean intake of TAC from supplements was 0.6 mmol/day compared to 0.8 mmol/day among cases. We found no appreciable differences between controls and cases regarding BMI, smoking habits, and intake of energy, calcium and zinc. Multivitamin, supplemental vitamin C and supplemental vitamin E use was more common among cases compared to controls and high grade cases used more multivitamins compared to controls. Intake of the major contributors to dietary TAC was similar across disease categories, except coffee and tea intakes that were slightly lower in high grade and fatal cases (Table 2).

**Table 1:** Age standardized study population characteristic by controls and cases in means (standard deviations) or percents, Cancer of the Prostate Sweden Study

	Controls	Cases
N	1112	1489
FRAP diet (mmol/d)	22.2 (8.6)	22.3 (8.4)
FRAP fruit/vegs (mmol/d)	3.5 (1.8)	3.5 (1.7)
FRAP suppl. (mmol/d)	0.6 (1.2)	0.8 (1.4)
Age	67.7 (7.5)	66.8 (7.3)
BMI	26(3)	26(3)
Current smokers (%)	12	11
Never smokers (%)	38	39
0–9 yrs education (%)	46	46
10–12 years education (%)	42	40
13+ yrs education (%)	11	14
Energy kcal/d)	2218 (655)	2283 (646)
Coffee (cups/day)	3.1 (2.0)	3.1 (1.9)
Tea (cups/day)	0.6 (1.0)	0.7 (1.0)
Chocolate (serv./d)	0.2 (0.2)	0.2 (0.3)
Berries (serv./d)	0.2 (0.2)	0.2 (0.2)
Boiled potatoes (serv./d)	0.6(0.4)	0.6(0.4)
Multivitamin use (%)	16	22
Vitamin C suppl. use (%)	12	15
Vitamin E suppl. use (%)	3	4
Calcium (mg/d)	1197 (363)	1199 (370)
Zinc (mg/d)	11.7 (1.8)	11.6 (1.9)
PSA at diagnosis		89 (360)

**Table 2:** Age standardized study population characteristic by subtypes of cases in means (standard deviations) or percents, Cancer of the Prostate Sweden Study

	Fatal	Advanced	Localized	High grade	Low grade
N	307	499	1020	231	693
FRAP diet (mmol/d)	21.6 (7.9)	22.5 (8.9)	22.2 (8.1)	21.3 (8.0)	22.3 (8.0)
FRAP fruit/vegs (mmol/d)	3.5 (1.9)	3.4 (1.7)	3.5 (1.7)	3.6 (2.0)	3.4 (1.6)
FRAP suppl. (mmol/d)	0.7 (1.2)	0.8 (1.4)	0.9 (1.4)	0.9 (1.3)	0.8 (1.4)
Age	67.0(7.3)	68.6 (7.4)	66.0 (7.1)	68.3 (7.4)	65.4 (7.0)
BMI	26 (3)	26 (3)	26 (3)	26 (3)	26 (4)
Current smokers (%)	14	13	10	12	10
Never smokers (%)	42	39	39	41	38
0–9 years education (%)	53	51	44	48	44
10–12 years education (%)	37	41	40	40	41
13+ yrs education (%)	10	8	17	13	16
Energy kcal/d)	2363 (701)	2334(681)	2270 (630)	2387(655)	2234 (624)
Coffee (cups/day)	3.1 (1.8)	3.2(2.0)	3.1 (1.9)	3.0 (1.9)	3.1 (1.8)
Tea (cups/day)	0.5 (0.8)	0.5 (0.9)	0.7 (1.1)	0.6 (0.8)	0.7 (1.1)
Chocolate (serv./d)	0.2 (0.3)	0.2 (0.3)	0.2 (0.2)	0.2 (0.2)	0.2 (0.2)
Berries (serv./d)	0.2 (0.3)	0.2 (0.2)	0.2 (0.3)	0.2 (0.3)	0.1 (0.2)
Boiled potatoes (serv./d)	0.6(0.4	0.6 (0.4)	0.6 (0.4)	0.6 (0.3)	0.6 (0.4)
Multivitamin use (%)	21	22	21	29	20
Vitamin C suppl. use (%)	12	15	15	16	14
Vitamin E suppl. use (%)	3	3	4	3	5
Calcium (mg/d)	1241(406)	1222 (390)	1188 (363)	1213 (381)	1193 (363)
Zinc (mg/d)	11.5 (1.9)	11.5 (1.9)	11.6 (1.9)	11.6 (1.9)	11.7 (1.8)
PSA at diagnosis	264 (573)	276 (665)	39 (241)	167 (364)	22 (136)

Fatal prostate cancer: Death from prostate cancer through follow up. Advanced prostate cancer: M1, N1, T3 or T4 and death during follow up. Localized prostate cancer: T1 or T2 and N0/M0. High-grade prostate cancer: Gleason sum 8–10. Low-grade prostate cancer: Gleason sum 2–6

### TAC and prostate cancer risk

Dietary TAC and TAC from fruit and vegetables only were not significantly associated with prostate cancer risk (Table 3). To investigate whether the extreme high and low intakes of TAC were associated with all incident prostate cancer we calculated odds ratios based on decile distribution for the exposure variables dietary TAC (OR = 1.03, 95 % CI 0.72–1.48 and TAC from fruit and vegetables (OR = 0.88, 95 % CI 0.61–1.26) respectively between lowest and highest decile of intake.

**Table 3:** Odds ratios, OR (and 95 % CI) of prostate cancer by quintiles of TAC all foods, TAC from fruits and vegetables, Cancer of the Prostate Sweden

FRAP food only	Q1	Q2	Q3	Q4	Q5	p-t rend
All prostate cancer	294/226	292/228	301/220	308/212	294/226	
Age and region adjusted OR	1.00	1.02 (0.80–1.31)	1.08 (0.84–1.39)	1.11 (0.87–1.43)	1.02 (0.79–1.31)	0.76
Fully adjusted OR	1.00	0.98(0.77–1.26)	1.05 (0.82–1.35)	1.09 (0.85–1.40)	1.05 (0.82–1.36)	0.53
Lethal prostate cancer	51/226	49/228	49/220	42/212	41/226	
Age and region adjusted OR	1.00	0.98 (0.63–1.51)	1.01(0.65–1.56)	0.91 (0.58–1.43)	0.89 (0.57–1.43)	0.58
Fully adjusted OR	1.00	0.98 (0.63–1.53)	0.98(0.63–1.53)	0.92 (0.58–1.45)	0.87 (0.55–1.39)	0.52
Advanced prostate cancer	94/226	103/228	95/220	102/212	105/226	
Age and region adjusted OR	1.00	1.11 (0.78–1.55)	1.04(0.74–1.47)	1.20 (0.85–1.68)	1.23 (0.88–1.73)	0.20
Fully adjusted OR	1.00	1.08 (0.77–1.53)	1.02(0.72–1.44)	1.18 (0.83–1.67)	1.23 (0.87–1.74)	0.21
Localized prostate cancer	201/226	196/228	216/220	212/212	195/226	
Age and region adjusted OR	1.00	1.01 (0.77–1.33)	1.16 (0.89–1.53)	1.11 (0.84–1.46)	0.96 (0.73–1.26)	0.90
Fully adjusted OR	1.00	0.96 (0.73–1.27)	1.12 (0.85–1.47)	1.09 (0.82–1.43)	1.00 (0.75–1.32)	0.80
High-grade prostate cancer	56/226	39/228	47/220	49/212	40/226	
Age and region adjusted OR	1.00	0.72 (0.46–1.13)	0.88(0.57–1.36)	0.99(0.64–1.52)	0.79(0.50–1.25)	0.64
Fully adjusted OR	1.00	0.68 (0.43–1.07)	0.83(0.53–1.28)	0.95(0.61–1.48)	0.78(0.49–1.24)	0.63
Low–grade prostate cancer	134/226	139/228	136/220	146/212	138/226	
Age and region adjusted OR	1.00	1.06 (0.78–1.44)	1.07 (0.78–1.45)	1.11 (0.81–1.50)	0.99 (0.72–1.35)	0.22
Fully adjusted OR	1.00	1.02 (0.75–1.39)	1.03 (0.76–1.41)	1.09 (0.80–1.49)	1.04 (0.76–1.42)	0.74
FRAP fruit & vegetables	Q1	Q2	Q3	Q4	Q5	p-trend
All prostate cancer	297/222	277/243	306/213	311/209	295/224	
Age and region adjusted OR	1.00	0.84(0.66–1.08)	1.06(0.82–1.36)	1.09(0.85–1.39)	0.97(0.76–1.25)	0.50
Fully adjusted OR	1.00	0.83(0.64–1.07)	1.04(0.80–1.34)	1.07(0.83–1.38)	0.94(0.73–1.22)	0.82
Lethal prostate cancer	53/222	33/243	52/213	45/209	48/224	
Age and region adjusted OR	1.00	0.57 (0.36–0.92)	1.01 (0.65–1.54)	0.91 (0.58–1.41)	0.91 (0.59–1.41)	0.74
Fully adjusted OR	1.00	0.61 (0.38–0.99)	1.13 (0.73–1.77)	1.07 (0.67–1.71)	1.02 (0.65–1.61)	0.39
Advanced prostate cancer	114/222	77/243	106/213	98/209	102/224	
Age and region adjusted OR	1.00	0.64 (0.44–0.94)	0.90(0.63–1.30)	0.92(0.64–1.33)	0.91(0.63–1.30)	0.76
Fully adjusted OR	1.00	0.65(0.46–0.93)	1.01(0.72–1.41)	1.01(0.71–1.42)	0.96(0.68–1.35)	0.49
Localized prostate cancer	192/222	204/243	205/213	219/209	199/224	
Age and region adjusted OR	1.00	0.93 (0.71–1.23)	1.09 (0.83–1.44)	1.15 (0.87–1.51)	1.00 (0.76–1.32)	0.67
Fully adjusted OR	1.00	0.90 (0.68–1.18)	1.04 (0.79–1.38)	1.07 (0.81–1.42)	0.94 (0.71–1.25)	0.95
High-grade prostate cancer	53/222	27/243	46/213	45/209	58/224	
Age and region adjusted OR	1.00	0.47(0.28–0.77)	0.88 (0.57–1.37)	0.89 (0.57–1.39)	1.08 (0.71–1.64)	0.12
Fully adjusted OR	1.00	0.49(0.30–0.82)	0.93 (0.59–1.46)	0.99 (0.62–1.57)	1.13 (0.73–1.76)	0.08
Low-grade prostate cancer	129/222	149/243	140/213	150/209	124/224	
Age and region adjusted OR	1.00	0.99 (0.73–1.34)	1.06 (0.78–1.45)	1.15 (0.85–1.57)	0.91 (0.66–1.24)	0.67
Fully adjusted OR	1.00	0.94 (0.69–1.28)	1.01 (0.73–1.38)	1.09 (0.80–1.50)	0.85 (0.62–1.18)	0.46

Fully adjusted models are adjusted for: age, region, smoking (never, former, current), BMI (categories), education (categories), and energy intake (categories)

We performed a stratified analysis of TAC intake from diet and fruit and vegetables in ever smokers and never smokers. No appreciable differences were observed compared to the total population. For dietary TAC and TAC from fruit & vegetables among ever smokers the odds ratios in the highest quintile compared to the lowest quintile was: 1.12, (95 % CI 0.87–1.65) and 0.95 (95 % CI 0.69–1.31) respectively. Furthermore among never smokers the odds ratios in the highest quintile compared with the lowest was for dietary TAC 0.69, (95 % CI 0.45–1.07) and TAC from fruit & vegetables 0.96, (95 % CI 0.63–1.48) respectively.

Supplement use was more common among cases compared to controls, and consequently, we found a positive association between highest level of TAC intake from supplements and total, advanced, localized, high grade and low grade prostate cancer (total: OR = 1.37, 95 % CI 1.08–1.73, advanced: 1.51, 95 % CI 1.11–2.06, localized: 1.36, 95 % CI 1.06–1.76, high grade: 1.60, 95 % CI 1.06–2.40, low grade: 1.36, 95 % CI 1.03–1.81).

The major contributors to TAC derived from supplements were multivitamins and vitamin C. We observed a positive association with use of the highest doses of multivitamins and total (OR = 1.27, 95 % CI: 1.01–1.60) and high grade prostate cancer risk (1.93, 95 % CI: 1.32–2.81) (Table 4), whereas no association was seen for vitamin C supplements. To investigate if the findings for multivitamin use could be the result of reverse causation, (i.e., men began taking supplements as a result of feeling poorly due to disease prior to diagnosis), we took into account the duration of supplement use (vitamin C and multivitamins) and divided subjects into four groups: Never users, short-term users (0–2 years), intermediate-term users (2–5 years) and long-term users (>5 years). We found a positive association for longer duration of multivitamin use and risk of advanced (OR = 1.56, 95 % CI 1.02–2.40) and high-grade prostate cancer (2.00, 95 % CI 1.20–3.36) respectively for long-term users versus never users. For vitamin C use, we found a significantly increased risk of total, advanced and fatal prostate cancer among long-term compared to never users (total: OR = 1.32, 95 % CI 1.04–1.66, advanced 1.52, 95 % CI 1.03–2.23, fatal 1.23, 95 % CI 1.06–2.02).

**Table 4:** Odds ratios, OR (and 95 % CI) of prostate cancer by major contributors to TAC: coffee, tea, berries, chocolate, boiled potatoes, multivitamins and vitamin C supplements, Cancer of the Prostate Sweden

Coffee	None	<2 cups/day	2–3cups/day	4–5cups/day	≥6 cups/day	p-trend
All prostate cancer	139/98	150/121	644/491	413/295	143/107	
Fully adj. OR	1.00	0.88(0.61–1.26)	0.93(0.69–1.26)	1.03(0.75–1.42)	0.95(0.64–1.40)	0.87
Fatal prostate cancer	14/44	32/175	103/491	68/295	15/107	
Fully adj. OR	1.00	0.58(0.31–1.07)	0.73(0.45–1.18)	0.87(0.52–1.44)	0.56(0.29–1.08)	0.51
Advanced prostate cancer	47/121	47/121	207/491	151/295	53/107	
Fully adj. OR	1.00	0.90(0.54–1.52)	0.92(0.60–1.41)	1.09(0.70–1.72)	1.00(0.58–1.71)	0.65
Localized prostate cancer	98/98	103/121	452/491	273/295	94/107	
Fully adj. OR	1.00	0.86(0.57–1.28)	0.98(0.70–1.36)	1.04(0.72–1.48)	0.93(0.60–1.44)	0.86
High grade prostate cancer	30/98	22/121	98/491	62/295	19/107	
Fully adj. OR	1.00	0.53(0.28–1.01)	0.55(0.33–0.92)	0.59(0.24–1.01)	0.45(0.22–0.90)	0.05
Low grade prostate cancer	55/98	79/121	313/491	177/295	69/107	
Fully adj. OR	1.00	1.22(0.77–1.94)	1.22(0.82–1.80)	1.21(0.79–1.84)	1.24(0.76–2.05)	0.60
Tea	None	<1cup/day	1–2 cups/day	>2 cups/day		
All prostate cancer	605/484	370/262	439/310	75/56		
Fully adj. OR	1.00	1.12(0.91–1.37)	1.09(0.89–1.34)	0.97(0.65–1.44)		0.98
Fatal prostate cancer	142/484	73/262	83/310	9/56		
Fully adj. OR	1.00	0.95(0.66–1.36)	0.90(0.64–1.26)	0.60(0.28–1.30)		0.16
Advanced prostate cancer	230/484	131/262	121/310	17/56		
Fully adj. OR	1.00	1.06(0.81–1.40)	0.86(0.64–1.15)	0.67(0.37–1.23)		0.12
Localized prostate cancer	390/484	251/262	318/310	61/56		
Fully adj. OR	1.00	1.15(0.92–1.45)	1.20(0.96–1.50)	1.12(0.74–1.70)		0.38
High grade prostate cancer	94/484	65/262	63/310	9/18		
Fully adj. OR	1.00	1.27(0.88–1.83)	0.92(0.63–1.36)	0.64(0.29(1.40)		0.17
Low grade prostate cancer	265/484	166/262	221/310	41/56		
Fully adj. OR	1.00	1.12(0.86–1.45)	1.25(0.97–1.62)	1.12(0.70–1.81)		0.33
Berries	None	1–3 /month	>1–3/month			
All prostate cancer	276/231	739/482	474/399			
Fully adj. OR	1.00	1.16(0.93–1.44)	0.90(0.70–1.44)			0.08
Fatal prostate cancer	67/231	129/482	610/399			
Fully adj. OR	1.00	0.95(0.67–1.37)	0.87(0.59–1.27)			0.47
Advanced prostate cancer	98/231	236/482	164/399			
Fully adj. OR	1.00	1.15(0.85–1.55)	0.90(0.65–1.25)			0.15
Localized prostate cancer	186/321	513/482	321/399			
Fully adj. OR	1.00	1.16(0.91–1.49)	0.89(0.68–1.16)			0.11
High grade prostate cancer	38/231	104/482	89/399			
Fully adj. OR	1.00	1.19(0.78–1.83)	1.10(0.70–1.72)			0.91
Low grade prostate cancer	133/231	370/482	190/399			
Fully adj. OR	1.00	1.17(0.89–1.53)	0.75(0.56–1.02)			<0.01
Chocolate	None	1–3/month	>1–3/month			
All prostate cancer	232/258	645/457	612/397			
Fully adj. OR	1.00	1.38(1.09–1.73)	1.43(1.12–1.82)			0.03
Fatal prostate cancer	59/258	119/457	129/397			
Fully adj. OR	1.00	1.26(0.86–1.85)	1.47(0.99–2.20)			0.09
Advanced prostate cancer	93/258	194/457	212/397			
Fully adj. OR	1.00	1.19(0.86–1.63)	1.40(1.01–1.96)			0.06
Localized prostate cancer	146/258	463/457	411/397			
Fully adj. OR	1.00	1.46(1.13–1.89)	1.43(1.08–1.88)			0.11
High grade prostate cancer	42/258	88/457	101/397			
Fully adj. OR	1.00	1.04(0.67–1.60)	1.20(0.77–1.88)			0.33
Low grade prostate cancer	102/258	310/457	281/397			
Fully adj. OR	1.00	1.38(1.03–1.85)	1.41(1.03–1.93)			0.09
Boiled potatoes	0–4×/w	4–6×/w	≥1×/day			
All prostate cancer	266/195	668/545	555/372			
Fully adj. OR	1.00	0.92(0.73–1.16)	1.12(0.87–1.42)			0.21
Fatal prostate cancer	42/195	151/545	114/372			
Fully adj. OR	1.00	1.27(0.85–1.90)	1.31(0.86–2.01)			0.26
Advanced prostate cancer	86/195	219/545	194/372			
Fully adj. OR	1.00	0.88(0.64–1.20)	1.07(0.77–1.49)			0.45
Localized prostate cancer	181/195	464/545	375/372			
Fully adj. OR	1.00	1.00(0.79–1.29)	1.20(0.92–1.57)			0.13
High grade prostate cancer	33/195	107/545	91/372			
Fully adj. OR	1.00	1.18(0.75–1.84)	1.33(0.83–2.12)			0.21
Low grade prostate cancer	134/195	107/545	91/372			
Fully adj. OR	1.00	0.90(0.68–1.19)	1.07(0.79–1.45)			0.45
Multivitamins	None	<7/week	≥7/week			
All prostate cancer	1166/930	69/32	254/150			
Fully adj. OR	1.00	1.48(0.95–2.30)	1.27(1.01–1.60)			0.07
Fatal prostate cancer	183/930	12/32	37/150			
Fully adj. OR	1.00	1.65(0.84–3.24)	1.37(0.95–1.99)			0.18
Advanced prostate cancer	391/930	24/32	84/150			
Fully adj. OR	1.00	1.69(0.97–2.97)	1.29(0.95–1.75)			0.08
Localized prostate cancer	803/930	46/32	171/150			
Fully adj. OR	1.00	1.34(0.83–2.17)	1.21(0.94–1.55)			0.18
High grade prostate cancer	165/930	14/32	52/150			
Fully adj. OR	1.00	2.20(1.11–4.33)	1.93(1.32–2.81)			0.03
Low grade prostate cancer	552/930	25/32	116/150			
Fully adj. OR	1.00	1.08(0.62–1.89)	1.25(0.85–1.85)			0.75
Vitamin C	None	<7/week	≥7/week			
All prostate cancer	1271/984	106/62	112/66			
Fully adj. OR	1.00	1.20(0.86–1.68)	1.22(0.88–1.70)			0.14
Fatal prostate cancer	200/984	19/62	13/66			
Fully adj. OR	1.00	1.27(0.75–2.16)	0.86(0.47–1.55)			0.19
Advanced prostate cancer	423/984	36/62	40/66			
Fully adj. OR	1.00	1.32(0.85–2.06)	1.47(0.96–2.25)			0.14
Localized prostate cancer	866/984	78/62	76/66			
Fully adj. OR	1.00	1.25(0.87–1.79)	1.15(0.80–1.65)			0.20
High grade prostate cancer	195/984	21/62	15/66			
Fully adj. OR	1.00	1.48(0.86–2,55)	0.93(0.50–1.71)			0.80
Low grade prostate cancer	593/984	43/62	57/66			
Fully adj. OR	1.00	0.99(0.65–1.51)	1.25(0.85–1.85)			0.18

Fully adjusted models are adjusted for: age, region, smoking (never, former, current), BMI (categories), education (categories), energy intake, calcium (quartiles), zinc(quartiles). Food models mutually adjusted for chocolate (categories), coffee (categories), berries (categories), tea (categories), boiled potatoes (categories). Supplement models mutually adjusted for vitamin C supplement intake and multivitamin intake

### Components of dietary TAC and prostate cancer

For the main contributors to dietary TAC intake there were some notable associations with prostate cancer (Table 4). As previously published for this population, coffee intake was inversely associated with risk of fatal and high grade prostate cancer [15]. Following mutual adjustment for the other contributors to TAC we found an inverse association between coffee intake and fatal and high grade prostate cancer with 45–55 % lower OR for fatal and high grade prostate cancer, comparing the highest category of intake (≥6cups/day) to those in the lowest category of intake. For tea intake we observed a similar association, although weaker and based on fewer participants with high intakes. For berry intake we saw no significant inverse association. For chocolate intake, there was a positive association with total, advanced, localized and low grade prostate cancer (total OR = 1.43, 95 % CI 1.12–1.82, advanced 1.40, 95 % CI 1.01–1.96, localized 1.43, 95 % CI 1.08–1.88, and low grade 1.41, 95 % CI 1.03–1.93). For boiled potatoes, there was no significant trend across categories of intake.

## Discussion

In this large Swedish population-based study, we found no convincing association between TAC from all foods or from fruit and vegetables only and overall prostate cancer. Other studies examining the risk of prostate cancer with a combined score of antioxidants and pro oxidants, also reported null results [1, 16]. The assessment of the total dietary antioxidant capacity captures both all known and unknown antioxidants, which should be more informative than analyses of single antioxidants. Studies of total antioxidant intake utilizing the FRAP assay have been negative for colorectal and endometrial cancer. But for gastric cancer, an inverse association has been documented between high intake of antioxidants from plant foods assessed by the FRAP assay and disease risk [17–20].

One possible reason for the lack of overall association for TAC intake from diet and prostate cancer in our study may be that some of the main contributors to the antioxidant intake had opposite associations with prostate cancer risk. Coffee, chocolate and berries were among the five major contributors, but as chocolate intake was positively associated with total, advanced, localized and low grade prostate cancer risk, the intake of coffee and berries seemed to be inversely associated with some of the disease sub-types.

TAC from coffee was suggestively associated with reduced risk for fatal and high grade cancer, which is in line with other population based studies [6, 15, 21, 22]. Recently several meta analyses have shown an inverse association between coffee consumption and prostate cancer, especially fatal and high grade cancers [23–26]. However, the composition of the diet with respect to TAC intake was different in the CAPS study compared to the Health Professionals Follow-up Study (HPFS) in the U.S. In the former study coffee contributed to 62 % of dietary TAC intake but only 28 % in the latter. The coffee intake in Sweden is considerably higher than in the US. Swedish per capita coffee consumption is 7.14 kg/year compared to the US with only 4.24 kg /year (2011) [27]. This may also reflect that overall total antioxidant intake in Sweden is somewhat higher compared with other populations: Among cases and controls in this study the daily average intake (energy adjusted) was 22.3 mmol/d and 22.2 mmol/d respectively. In the US population Health Professionals Follow up Study, the daily average intake of TAC from diet was 10.8 mmol/d [8]. In an Italian Study population the mean levels of total antioxidant intake from diet was 11.45 mmol/d utilizing the FRAP assay, but the authors did not include coffee in their calculation which may have resulted in an underestimation of the overall intake [28]. Other studies have used different units to quantify the antioxidant intake, making comparisons difficult [29].

The proportion of patients diagnosed with a higher clinical stage (T3, T4, N1 or M1) in the CAPS study was almost twice the proportion in the HPFS (33 % vs. 16 %) probably due to more extensive PSA screening in the U.S.

The observed association with chocolate could be biologically plausible, because the high amount of carbohydrates in chocolate could stimulate IGF-1 production as a possible mechanism contributing to increased tumor growth and a more malignant behavior of the disease [30, 31]. In one study, sucrose intake and disaccharides was positively associated with prostate cancer risk [32]. However two large prospective studies found no association between glycemic index and prostate cancer risk [33, 34]. On the other hand, dark chocolate is also high in flavonoids, a potent group of antioxidants [12], but there was no distinction between different types of chocolate in the CAPS questionnaire. The most likely explanation for this association, however is that it is a chance finding.

Because berries are a rich source of flavonoids, the trend indicating an inverse association with low-grade prostate cancer is plausible. Flavonoids are antioxidants that may inhibit the matrix metalloproteinase system (MMP) [35, 36], which is probably important in the invasive and metastatic process [37]. The 25 % reduction of risk for low-grade prostate cancer observed for those consuming berries more than 1–3 times per month could possibly be due to this chemopreventive effect. This novel finding is interesting due to a plausible mechanism of action. If a protective effect from substances abundant in berries exists – it would most likely be weak and mostly effective in the indolent, low-grade cases whereas an effect in high-grade cases would not be expected. In the CAPS questionnaire intake of all berries were aggregated into one question. This makes it difficult to determine whether the association was related to specific berries/phytochemicals or confounded by healthy lifestyle related behaviors. Furthermore, when we analyzed TAC from fruit and vegetables, where berries comprised 23 % of the TAC contribution, we observed no association with prostate cancer.

TAC derived from supplements appeared positively associated with all categories of prostate cancer, except fatal cancers. The risk estimates are in line with those previously found in the Health Professionals Follow-up Study [8]. However, we cannot rule out reverse causation, because men who experienced early symptoms of prostate cancer before diagnosis might have self-medicated with supplements. High doses of antioxidants taken as supplements may also have pro-oxidant properties [38, 39], that promote survival of malignant cells [40]. Recently, it was also shown in animal models, that dietary antioxidants may interfere with endogenous antioxidants by affecting feedback mechanisms [41]. All these explanations fits well with the observation that most nutrients follow a non-linear, inverted U-shaped curve with respect to their physiological function, and hence both deficiencies and high levels may be associated with disease [42]. Possible explanations for the differences between this population and the HPFS may be that almost twice as many individuals were regular supplement users in the US study population compared to the participants in CAPS. The long follow up time with multiple exposure assessments and large numbers cases with lethal disease in the HPFS can also explain why this finding was more apparent in the HPFS, compared to the fatal disease studied in CAPS.

In the present study we also had the ability to look more closely at the major contributors to TAC from supplements. The associations were mainly accounted for by multivitamin use, in line with findings in some other studies [43, 44], although others reported null results [45, 46]. However, in our study, both analyses on TAC from supplements and multivitamins must be interpreted with caution due to the limited number of supplement users and the possibility of reverse causation.

We are aware of other limitations in this study. Selection bias is a concern in case–control studies, as participating controls may be more health conscious than the general population. In our study, however, the groups were well balanced with regard to baseline characteristics. In addition, such selection bias would not explain our supplement results because dietary supplement use is associated with more health conscious behavior, Recall bias is another concern, because diet was assessed after the prostate cancer diagnosis which could give rise to differential misclassification of exposure. Reverse causation, especially in the assessment of supplement use as mentioned above, may explain the findings associated with TAC from supplements, since a proportion of the cases may have experienced disease-related symptoms such as fatigue and started using supplements due to this before the time of diagnosis. Multiple testing is always a concern when assessing many different outcomes. The lack of association between TAC from diet and prostate cancer risk could also in part be explained by the fact that, the FRAP assay only measure in vitro antioxidant activity and, does not take into account the diversity in bioavailability of different antioxidants and their in vivo metabolism.

There are several strengths to this study. The population-based design with a large number of controls and cases, and information on disease characteristics makes it possible to study not only total prostate cancer, but also sub-groups of the disease. The large amount of data collected, including information on diet and other lifestyle-related habits, makes it possible to adjust for many possible confounding factors. The measurement of the total antioxidant capacity in diet captures both all known and unknown antioxidants, which may strengthen the associations compared to examining only single antioxidants.

## Conclusion

In conclusion, we found no association between of total antioxidant capacity and prostate cancer, either from the total diet or from fruit and vegetables. High intake of antioxidants from supplements was associated with increased risk for all subgroups of prostate cancer, except fatal cancer. This finding must be interpreted with caution due to the possibility for reverse causation.

## Abbreviations

CAPS, Cancer of the Prostate in Sweden; FFQ, food frequency questionnaire; FRAP: ferric reducing antioxidant potential; HPFS, Health Professionals Follow-up Study; OR, odds ratio; TAC, total antioxidant capacity

## Additional files

Additional file 1: FRAP values. Description: Supplementary table of FRAP values from food, beverages and dietary supplements unique for the Scandinavian market, and not included in antioxidant food table. (DOCX 119 kb)
