# Supplementary material for: Total antioxidant intake and prostate cancer in the Cancer of the Prostate in Sweden (CAPS) study. A case control study
Source: BMC Cancer. 2016 Jul 11;16:438. doi: 10.1186/s12885-016-2486-8 (PMC4939657; doi:10.1186/s12885-016-2486-8)
Supplement: Additional file 1: — FRAP values. Description: Supplementary table of FRAP values from food, beverages and dietary supplements unique for the Scandinavian market, and not included in antioxidant food table. (DOCX 119 kb) [file 12885_2016_2486_MOESM1_ESM.docx]

FRAP values from food, beverages, dietary supplements unique for Scandinavian market and not available in the antioxidant food table

| **Product** | **FRAP (mmol/100g)** | **Date analyzed** | **Comment** |
| --- | --- | --- | --- |
| Cooked/smoked ham | 1.179 | 6/28/10 | Procured in Norway |
| Borg Light beer | 0.114 | 6/10/10 | Procured in Norway |
| Blood pudding | 0.162 | 6/30/10 | Shipped from Sweden |
| Vitamin C, supplement | 275.492 | 7/8/10 | Shipped from Sweden |
| C-Vitamin,Max Medica (Vitamin C, supplement) | 244.393 | 7/8/10 | Shipped from Sweden |
| Enomdan (Multivitamin) | 141.105 | 7/8/10 | Shipped from Sweden |
| Liver pate | 0.627 | 6/9/10 | Procured in Norway |
| Light Mayonnaise) | 0.220 | 6/9/10 | Procured in Norway |
| E-vimin 2_Propanol* (Vitamin E, supplement) | 0.071 | 7/19/10 | Shipped from Sweden |
| E-vitamin 2-Propanol*(Vitamin E, supplement) | 279.323 | 7/19/10 | Shipped from Sweden |
| Familj,ACO (Multivitamin) | 74.746 | 7/8/10 | Shipped from Sweden |
| Cooked/smoked ham | 0.460 | 6/28/10 | Procured in Norway |
| Cooked ham | 0.376 | 6/28/10 | Procured in Norway |
| Blood pudding original Swedish | 0.176 | 6/30/10 | Shipped from Sweden |
| Gilde fresh liver pate | 0.878 | 6/9/10 | Procured in Norway |
| Gilde cooked ham | 0.443 | 6/10/10 | Procured in Norway |
| Gilde smoked ham | 0.365 | 6/10/10 | Procured in Norway |
| Grillstad fresh liver pate | 0.284 | 6/9/10 | Procured in Norway |
| Hansa Light beer | 0.127 | 6/10/10 | Procured in Norway |
| Hervik red bilberry jam | 2.155 | 6/28/10 | Procured in Norway |
| ICA blood pudding | 0.124 | 6/30/10 | Shipped from Sweden |
| ICA cooked ham | 0.479 | 6/18/10 | Procured in Norway |
| Kalcipos (Calcium/vitamin D supplement) | 0.002 | 7/8/10 | Shipped from Sweden |
| Kavli caviar | 0.312 | 6/28/10 | Procured in Norway |
| Kavli cream cheese with ham | 0.047 | 6/10/10 | Procured in Norway |
| Møllerens bran | 0.502 | 6/10/10 | Procured in Norway |
| Regal(AXA) bran | 0.623 | 6/10/10 | Procured in Norway |
| Mills caviar | 0.365 | 6/9/10 | Procured in Norway |
| Mills majones,light mayonnaise | 0.343 | 6/9/10 | Procured in Norway |
| MittVal (Multivitamin) | 18.463 | 7/8/10 | Shipped from Sweden |
| Hash | 0.106 | 6/30/10 | Shipped from Sweden |
| Selenium | 0.019 | 7/12/10 | Shipped from Sweden |
| X-tra Cream chesse with ham | 0.033 | 6/10/10 | Procured in Norway |
| Stabburet caviar | 0.769 | 6/9/10 | Procured in Norway |
| Synnøve diet cheese | 0.013 | 6/10/10 | Procured in Norway |
| Jarlsberg,16% diet cheese | 0.022 | 6/18/10 | Procured in Norway |
| Tine Norvegia,16% diet cheese | 0.026 | 6/18/10 | Procured in Norway |
| Red bilberry jam | 1.574 | 6/18/10 | Procured in Norway |
| Euro Shopper red bilberry jam | 1.270 | 6/18/10 | Procured in Norway |
| Vita hjertegod light mayonnaise | 0.256 | 6/9/10 | Procured in Norway |
| Aas lettøl light beer | 0.139 | 6/10/10 | Procured in Norway |
|  |  |  |  |
|  |  |  |  |
| * extracted in 2-propanol due to oil content. |  |  |  |
